# Supplementary material for: Application and Diagnostic Performance of Two-Dimensional Shear Wave Elastography and Liver Fibrosis Scores in Adults with Class 3 Obesity
Source: Nutrients. 2023 Dec 25;16(1):74. doi: 10.3390/nu16010074 (PMC10780854; doi:10.3390/nu16010074)
Supplement: Supplementary file 1 [file nutrients-16-00074-s001.zip › nutrients-2759767-supplementary.pdf]

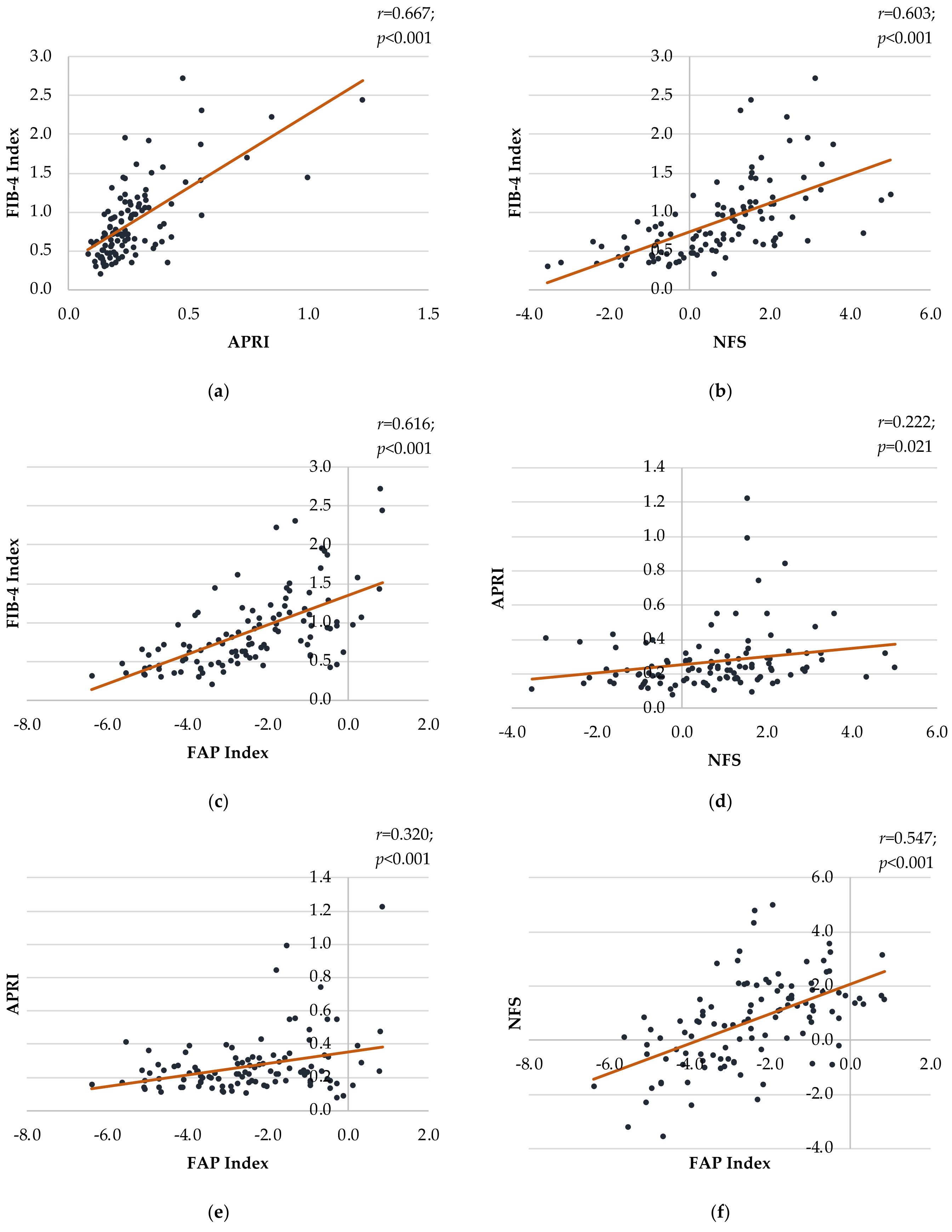

Supplementary Figure S1. Bland–Altman plot comparing non-invasive liver fibrosis screening tools in class 3 obesity. (**a**) FIB-4 Index and APRI; (**b**) FIB-4 Index and NFS; (**c**) FIB-4 Index and FAP Index; (**d**) APRI and NFS; (**e**) APRI and FAP Index; (**f**) NFS and FAP Index. APRI: AST (Aspartate Aminotransferase) to Platelet Ratio Index; EQI: ElastQ Imaging; FAP: Fibroblast Activation Protein; FIB-4: Fibrosis-4; NFS: NAFLD (Non-Alcoholic Fatty Liver Disease) Fibrosis Score.

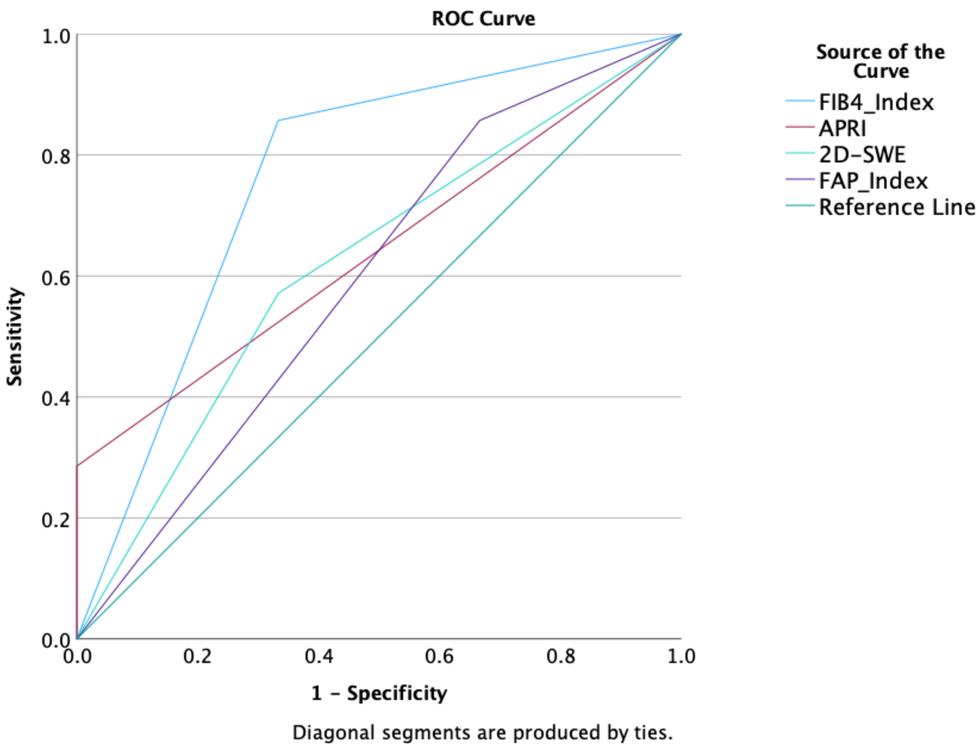

Supplementary Figure S2. Area under receiver operating characteristics curves (AUROC) of 2D-SWE, FIB-4 index, APRI, FAP index values for detecting risk of significant/advanced fibrosis (F ≥ 2). 2D-SWE: 2D-Shear Wave Elastography; APRI: AST (Aspartate Aminotransferase) to Platelet Ratio Index; FAP: Fibroblast Activation Protein; FIB-4: Fibrosis-4.
